# Prolactin-Stat5 signaling in breast cancer is potently disrupted by acidosis within the tumor microenvironment

**DOI:** 10.1186/bcr3467

**Published:** 2013-09-03

**Authors:** Ning Yang, Chengbao Liu, Amy R Peck, Melanie A Girondo, Alicia F Yanac, Thai H Tran, Fransiscus E Utama, Takemi Tanaka, Boris Freydin, Inna Chervoneva, Terry Hyslop, Albert J Kovatich, Jeffrey A Hooke, Craig D Shriver, Hallgeir Rui

**Affiliations:** 1 Department of Cancer Biology, Kimmel Cancer Center, Thomas Jefferson University, 233 South 10th Street, Philadelphia, PA 19107, USA; 2 Department of Pathology, Kimmel Cancer Center, Thomas Jefferson University, 233 South 10th Street, Philadelphia, PA 19107, USA; 3 Department of Medical Oncology, Kimmel Cancer Center, Thomas Jefferson University, 233 South 10th Street, Philadelphia, PA 19107, USA; 4 Department of Pharmaceutical Sciences, Thomas Jefferson University, Jefferson School of Pharmacy, 130 South 9th Street, Philadelphia, PA 19107, USA; 5 Department of Pharmacology and Experimental Therapeutics, Division of Biostatistics, Thomas Jefferson University, 1015 Chestnut Street, Philadelphia, PA 19107, USA; 6 MDR Global Systems, LLC, 425 Park Place, Windber, PA 15963, USA; 7 Department of Surgery, Walter Reed National Military Medical Center, 8901 Wisconsin Avenue, Bethesda, MD 20814, USA; 8 Department of Cancer Biology, Thomas Jefferson University, 233 South 10th Street, BLSB 330, Philadelphia, PA 19107, USA

**Keywords:** Breast cancer, Extracellular acidosis, Tumor microenvironment, Prolactin, Prolactin receptor, Stat5

## Abstract

**Introduction:**

Emerging evidence in estrogen receptor-positive breast cancer supports the notion that prolactin-Stat5 signaling promotes survival and maintenance of differentiated luminal cells, and loss of nuclear tyrosine phosphorylated Stat5 (Nuc-pYStat5) in clinical breast cancer is associated with increased risk of antiestrogen therapy failure. However, the molecular mechanisms underlying loss of Nuc-pYStat5 in breast cancer remain poorly defined.

**Methods:**

We investigated whether moderate extracellular acidosis of pH 6.5 to 6.9 frequently observed in breast cancer inhibits prolactin-Stat5 signaling, using *in vitro* and *in vivo* experimental approaches combined with quantitative immunofluorescence protein analyses to interrogate archival breast cancer specimens.

**Results:**

Moderate acidosis at pH 6.8 potently disrupted signaling by receptors for prolactin but not epidermal growth factor, oncostatin M, IGF1, FGF or growth hormone. In breast cancer specimens there was mutually exclusive expression of Nuc-pYStat5 and GLUT1, a glucose transporter upregulated in glycolysis-dependent carcinoma cells and an indirect marker of lactacidosis. Mutually exclusive expression of GLUT1 and Nuc-pYStat5 occurred globally or regionally within tumors, consistent with global or regional acidosis. All prolactin-induced signals and transcripts were suppressed by acidosis, and the acidosis effect was rapid and immediately reversible, supporting a mechanism of acidosis disruption of prolactin binding to receptor. T47D breast cancer xenotransplants in mice displayed variable acidosis (pH 6.5 to 6.9) and tumor regions with elevated GLUT1 displayed resistance to exogenous prolactin despite unaltered levels of prolactin receptors and Stat5.

**Conclusions:**

Moderate extracellular acidosis effectively blocks prolactin signaling in breast cancer. We propose that acidosis-induced prolactin resistance represents a previously unrecognized mechanism by which breast cancer cells may escape homeostatic control.

## Introduction

Extracellular acidosis is a frequent feature of the microenvironment in solid tumors, and acidosis is considered one of the major selective forces that promote evolution of aggressive and drug-resistant tumor clones [1]. Enhanced glycolysis with lactacidosis is a key contributor to reduced extracellular pH (pH_e_) in tumors [2]. *In vivo* measurements have revealed pH_e_ values of 6.5 to 6.9 in human breast cancer and other malignant tumors compared to normal tissue pH_e_ values of 7.2 to 7.4 [3,4]. Cancer cell glycolysis may be anaerobic as a consequence of hypoxia (the Pasteur effect) or aerobic due to metabolic reprogramming even under normoxic conditions (the Warburg effect) [5]. In addition, glycolysis in cancer-associated fibroblasts may contribute to extracellular acidosis in the tumor microenvironment [6]. Importantly, extracellular acidosis of malignant tumors potentiates cancer progression by facilitating tumor invasion [7,8], suppressing immune responses [9] and promoting metastasis in mouse models [10,11]. Elevated lactic acid secretion and acidosis were also associated with higher incidence of metastases in various human cancers [12,13]. However, the molecular mechanisms underlying selection for more aggressive cancer clones during acidosis remain incompletely understood and may vary between cancer types.

In breast cancer, prolactin has been implicated as a tumor promoter based on experimental studies in rodents [14,15] and the association between elevated circulating prolactin levels and increased risk of developing breast cancer [16]. Prolactin sustains nuclear tyrosine phosphorylated Stat5 (Nuc-pYStat5) and supports survival and expansion of differentiated luminal breast epithelial cells [17,18] and maintains their sensitivity to cell death [19]. In breast cancer cell lines, experimental activation of Stat5 promotes differentiation, inhibits invasive characteristics [20-22], and blocks progesterone-induced emergence of a drug-resistant CK5-positive cell population [23] with tumor-initiating characteristics [24-26]. In clinical breast cancer specimens, loss of Nuc-pYStat5 is associated with poor prognosis and increased risk of tamoxifen resistance [27-30]. Thus, a dual role of prolactin-Stat5 signaling in breast cancer has been proposed, wherein initial pathway activation promotes cell survival and tumor formation, whereas differentiation-promoting effects of prolactin-Stat5 signaling may support homotypic adhesion and suppress subsequent invasive behavior and progression [31]. However, little is known about the molecular causes for frequent loss of Stat5 tyrosine phosphorylation in human breast cancer.

Intriguingly, surface plasmon resonance studies have shown that prolactin interaction with its receptor is disrupted at pH of 6.0 or lower [32]. Such low pH occurs in early endosomes and in prolactin secretory vesicles of pituitary lactotrophs and may facilitate recycling of prolactin receptors and reversible prolactin aggregation, respectively [32]. Although pH_e_ lower than 6.5 rarely occurs in extracellular space of solid tumors, it has remained unclear, based on limited *in vitro* experiments [32-34], whether such moderate extracellular acidosis of the microenvironment of breast cancer affects prolactin signaling. Based on *in vitro* and *in vivo* experimental approaches and extensive quantitative *in situ* analyses of human breast cancer specimens, we now demonstrate that prolactin activation of prolactin receptors is selectively disrupted even at mildly acidic pH_e_ of 6.8. The new observations identify acidosis as a significant contributor to loss of Nuc-pYStat5 in clinical breast cancer specimens, and implicate acidosis-induced prolactin resistance as a previously unrecognized mechanism by which breast cancer cells may evade homeostatic control.

## Methods

### Cell culture, reagents and antibodies

T47D, SKBR3, MDA-MB-468, MCF-7 and BT474 cells (ATCC, Manassas, VA, USA) were cultured as previously described [35]. Mouse promyeloid 32D cells stably transfected with human prolactin receptors (32D-hPrlR cells) or human growth hormone (GH) receptors (32D-hGHR cells), were cultured as previously described [36]. Recombinant human prolactin and human GH were purchased from Dr. A. F. Parlow under the sponsorship of the National Hormone and Pituitary Program. Epidermal growth factor (EGF) was from Peprotech (Rocky Hill, NJ, USA). Monoclonal anti-pY-Stat5 (AX1) and rabbit antisera to Jak1 and Stat3, Stat5a and Stat5b were provided by Advantex BioReagents (Houston, TX, USA). Monoclonal Stat5, Jak1 and Erk antibodies were from BD Transduction Laboratories (Lexington, KY, USA). Monoclonal Jak2 antibody was from Biosource (Camarillo, CA, USA). Mouse monoclonal antibodies to phosphothreonine/tyrosine-ERK1/2 and phosphotyrosine-Stat3 were from Cell Signaling Technology (Beverly, MA, USA). Polyclonal antibody anti-Stat5 was from Santa Cruz Biotechnology (Santa Cruz, CA, USA). Rabbit anti-JAK2 antibody and mouse anti-phosphotyrosine antibody (4G10) were from Millipore (Billerica, MA, USA). Monoclonal antibodies used for immunohistochemistry include anti-pancytokeratin, anti-estrogen receptor (ER) (1D5), anti-progesterone receptor (PR) (PgR636) and antiKi67 (MIB-1) from Dako (Carpinteria, CA, USA), pY-Stat5 antibody (Epitomics, Burlingame, CA, USA), anti-glucose transporter 1 (GLUT1) from Thermo Fisher Scientific (Fremont, CA, USA), anti-PrlR (ECD) (1A2B1; Invitrogen, Carlsbad, CA, USA) and anti-lactate dehydrogenase-5 (LDH5) (Abcam, Cambridge, CA, USA).

### Three-dimensional cell culture

To generate three-dimensional spheroids of T47D cells, one million cells were loaded into a rotating bioreactor (Rotary Cell Culture System; Synthecon, Houston, TX, USA) and cultured at 7 to 8 rpm in buffered RPMI supplemented with 10% fetal calf serum (FCS) at 37°C for 36 h in a CO2 incubator. Spheroids (1 to 2 mm in diameter) were collected and transferred to serum-free medium with or without prolactin at pH 6.8 or 7.4 as indicated. Spheroids were then immediately formalin-fixed and paraffin-embedded.

### Cell stimulation, protein extraction, immunoprecipitation, and immunoblotting

For breast cancer cell lines, confluent cells were serum-starved overnight in culture medium, then incubated in fresh buffered RPMI (supplemented with 25 mM HEPES and 35 mM MOPS, adjusted to desired pH) for 15 min at 37°C before stimulation. Hormone or growth factor treatments were carried out at 37°C for 15 min, unless otherwise specified. 32D-hPrlR and 32D-hGHR cells were serum-starved for 5 h before stimulation. Cell stimulation, protein extraction, immunoprecipitation and immunoblotting were performed as described [35]. Briefly, for detection of pYStat5, pYJak1 or pYJak2, cell lysates were first immuoprecipitated with anti-Stat5, anti-Jak1 or anti-Jak2 antibodies and proteins were separated by SDS-PAGE. Immunoblotting with anti-pYStat5 for detecting pY-Stat5 or anti-phosphotysine (4G10) for detecting pYJak1 and pYJak2 was then conducted. For detection of other proteins whole cell lysates were used in western blotting.

### Quantitative RT-PCR

qRT-PCR assays were performed with RNA isolated from SKBR3 cells using RNeasy kit (Qiagen, Venlo, Netherlands). cDNA was generated using Iscript (Bio-Rad, Hercules, CA, USA) and subjected to quantitative PCR using forward/reverse primers CISH-f/r(CTGCTGTGCATAGCCAAGAC/GTGCCTTCTGGCATCTTCTG) and c-JUN-f/r(CACGTTAACAGTGGGTGCCA/CCCCGACGGTCTCTCTTCA).

### T47D xenograft tumors, prolactin treatment and tumor pH_e_ measurement *in vivo*

T47D xenotransplants were grown as previously described [36]. Animal studies reported here adhered to international guidelines of ethical conduct and were approved by Thomas Jefferson University Institutional Animal Care and Use Committee under protocol 789D to HR. Briefly, 5- to 7-week-old female nude mice implanted with 17β-estradiol pellets (0.72 mg; Innovative Research of America, Sarasota, FL, USA) were injected subcutaneously with 5 × 10^6^ T47D cells into two flank sites. For *in vivo* prolactin treatment, tumor-bearing mice were injected intraperitoneally with either vehicle control (*n* = 3) or 1 μg/g body mass of human prolactin (*n* = 3). One h after injection, mice were euthanized and tumors collected. The *in vivo* prolactin treatment experiment was independently repeated a second time. *In vivo* tumor pH measurements were performed individually on a total of nine anesthetized T47D tumor-bearing mice by inserting a miniature pH electrode (IC 501; Samuel Agulian, Hamden, CT, USA) 2 to 4 mm into the tumor through a small skin incision. *In vivo* intraperitoneal pH measurement was carried out in parallel in three of the same anesthetized T47D tumor-bearing mice by inserting the micro pH electrode into the peritoneal cavity through a skin incision. The mice were euthanized after the procedures.

### Breast tumor specimens

Two cohorts of archival and de-identified formalin-fixed, paraffin-embedded breast cancer specimens were analyzed under Thomas Jefferson University Institutional Review Board approved protocol 09G.355. Cohort I was from Walter Reed National Military Medical Center (Bethesda, MD, USA) and represented whole tissue sections from patients with invasive carcinomas. Histology, ER/PR, Ki67 and PrlR were evaluated by a pathologist. Cohort II was a breast cancer progression array of specimens from Thomas Jefferson University (Philadelphia, PA, USA) constructed by cutting edge matrix assembly [37], comprising 40 healthy breast tissues and 140 breast carcinoma specimens, including ductal carcinoma *in situ*, primary invasive ductal carcinomas (grades 1 to 3), and lymph node metastases [38].

### Immunohistochemistry (IHC) and quantification

IHC and automated quantitative analysis (AQUA) were performed as described previously [38]. Briefly, fluorescent images of stained slides were captured in three channels (FITC/Alexa-488, Cy5, or DAPI). AQUA scores for pYStat5 and GLUT1 represent average signal intensity within the epithelial cell population as defined by cytokeratin positivity. Cell-based quantitative analysis was performed using Tissue Studio (Definiens, Parsippany, NJ, USA).

### Densitometric and statistical analysis

Immunoblots were scanned and densitometric quantification of images exposed in the linear range was performed using Image J (NIH, Bethesda, MD, USA). Data from at least three experiments are presented as mean ± standard error (SE). Statistical significance of differences was estimated by *t* test or analysis of variance (ANOVA). Association between Nuc-pYStat5 and GLUT1 was evaluated in Cohort I and II of patient-derived specimens. For AQUA, quantification of Nuc-pYStat5 and cytoplasmic GLUT1 were obtained at whole tissue, regional, and cellular levels of Cohort I. Based on discrete distribution of the AQUA score, Nuc-pYStat5 scores above 65^th^ percentile of whole tissue level were defined as high and GLUT1 scores above 83^rd^ percentile of whole tissue level were defined as positive. At the whole tissue level, Fisher’s exact test was used to analyze association between Nuc-pYStat5 levels (high vs. low) and other tumor variables. Because multiple spots from each tumor section were used to obtain regional information, analyses at the regional level employed the generalized linear mixed-effects model to model levels of Nuc-pYStat5 with the random effect of slide and fixed effect of GLUT1. For Tissue Studio analyses, quantification of Nuc-pYStat5 and GLUT1 levels at the cellular level, corresponding percentile cut points were used to partition the staining levels into high and low. Cellular Nuc-pYStat5 levels were analyzed in logistic regression model with GLUT1 levels as predictor. Data were analyzed in R 2.14 (R Foundation for Statistical Computing, [39]) and SAS 9.3 (SAS Institute Inc., Cary, NC, USA).

## Results

### Prolactin activation of Stat5 in human breast cancer cell lines is disrupted by moderate extracellular acidosis

**Figure 1:**
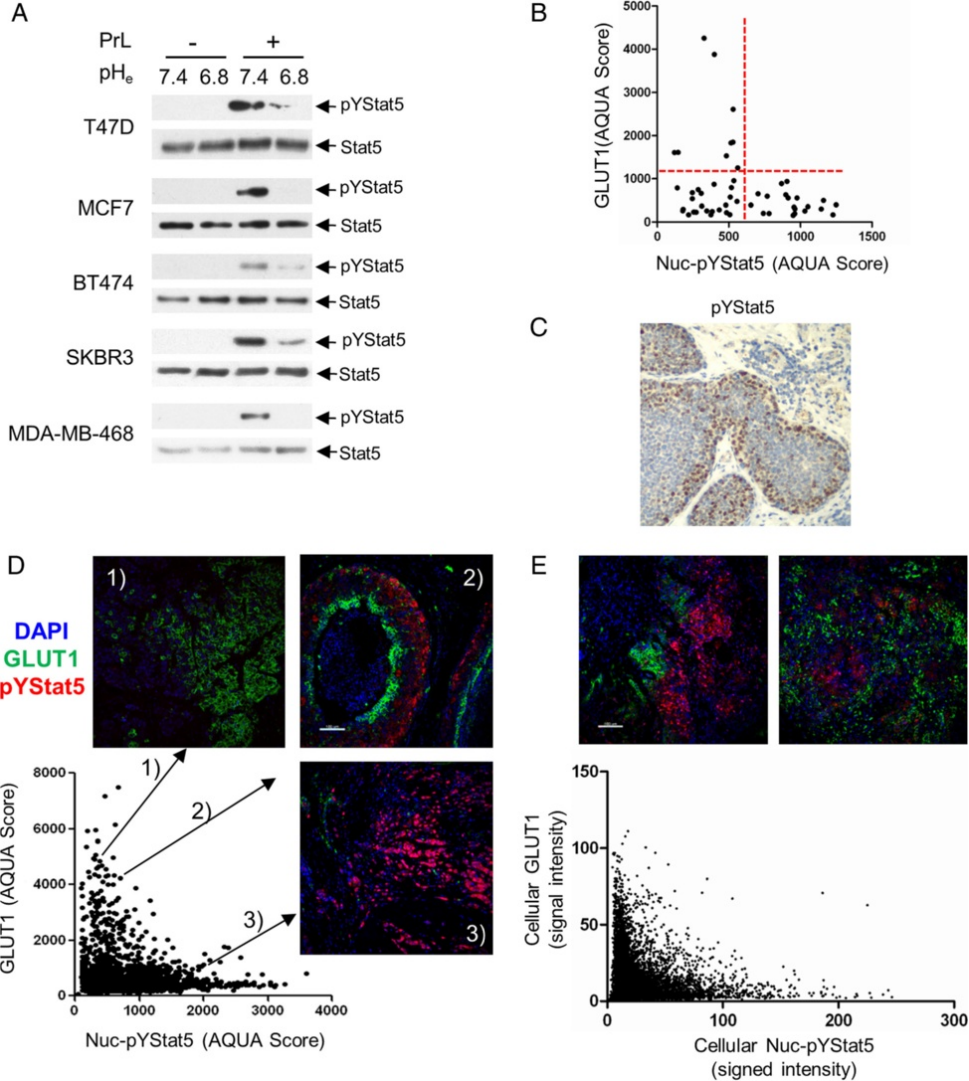
**Mutually exclusive expression of nuclear-localized pYStat5 and GLUT1 in human breast cancer. (A)** Five human breast cancer cell lines were treated with prolactin or vehicle for 15 min at pH_e_ 7.4 or pH_e_ 6.8. Cell lysates were immunoprecipitated with anti-Stat5, resolved by SDS-PAGE and immunoblotted with anti-pYStat5 or anti-Stat5. **(B)** Median GLUT1 and Nuc-pYStat5 levels (AQUA scores) of the 52 breast cancer specimens in Cohort I are shown. Red dashed lines indicate the cutoff values for GLUT1 positivity and Nuc-pYStat5 positivity. **(C)** Example of a human invasive ductal carcinoma showing regional variability of pYStat5 IHC staining intensity. **(D)** GLUT1 and Nuc-pYStat5 AQUA scores of 2,244 randomly sampled tumor regions from 52 breast cancer specimens of Cohort 1. Examples of co-staining images show high GLUT1/low Nuc-pYStat5 (panel 1), high GLUT1/high Nuc-pYStat5 (panel 2), or low GLUT1/low Nuc-pYStat5 (Panel 3). **(E)** Cellular GLUT1 and Nuc-pYStat5 intensities in 8,804 cells sampled from six tumor spots displaying both high GLUT1 and high Nuc-pYStat5 are scatter-plotted. Representative images used for the analysis are shown. AQUA, automated quantitative analysis; GLUT1, glucose transporter 1; IHC, immunohistochemistry; Nuc-pYStat5, nuclear localized and tyrosine phosphorylated Stat5; pH_e_, extracellular pH.

Prolactin responsiveness of five human breast cancer cell lines, including ER-positive T47D, MCF7 and BT474 and ER-negative SKBR3 and MDA-MB-468, was analyzed at pH_e_ of 6.8 and at normal tissue pH_e_ of 7.4. Each of the five cell lines responded to prolactin by inducible tyrosine phosphorylation of Stat5 at normal tissue pH_e_ of 7.4, whereas prolactin-induced Stat5 phosphorylation was abolished or nearly abolished at pH_e_ 6.8 (Figure 1A). We conclude that prolactin-induced Stat5 activation in human breast cancer cell lines is highly sensitive to moderate pH reduction.

### Mutually exclusive expression of nuclear localized, tyrosine phosphorylated Stat5 and GLUT1 in clinical human breast cancer specimens

To establish initial clinical relevance for the *in vitro* observations, we tested the hypothesis that levels of nuclear localized and tyrosine phosphorylated Stat5 (Nuc-pYStat5) would be low in human breast cancer specimens expressing elevated levels of GLUT1, a glucose transporter. GLUT1 is upregulated in breast carcinoma cells with elevated glycolytic metabolism and lactic acid production [40]. Whole tumor tissue sections from a cohort of 52 invasive breast cancer specimens were analyzed using the multiplexed and immunofluorescence-based AQUA platform [29,38]. Indeed, Nuc-pYStat5 status was negatively associated with GLUT1 status (Fisher’s exact test, *p =* 0.009), but not with levels of PrlR, ER/PR expression, histological classification (ductal vs. lobular), or proliferation index Ki67 (Table 1). While ER/PR-negative tumors were associated with positive GLUT1 expression (*p* <0.01), Nuc-pYStat5 status was not significantly associated with ER/PR status.

**Table 1:** Relationship between levels of Nuc-pYStat5 and other tumor variables in Cohort I

		**Nuc-pYStat5 *n *** **(%)**		**Fisher’s exact test, *P***
		**High**	**Low**	
GLUT1	High	0 (0)	9 (100)	0.009
	Low	20 (46.5)	23 (53.5)	
PrlR	High	9 (45)	11 (55)	0.561
	Low	11 (34.4)	21 (65.6)	
Ki67	> = 15%	11 (35.5)	20 (64.5)	0.770
	<15%	9 (42.9)	12 (57.1)	
Histology	IDC	18 (39.1)	28 (60.9)	0.784
	ILC	2 (50)	4 (50)	
ER/PR	Positive	18 (45.0)	22 (55.0)	0.100
	Negative	2 (16.7)	10 (83.3)	
**Total**		**20 (38.5)**	**39 (61.5)**	**52**

*Positive (ER/PR) status means ER or PR is positive, whereas negative ER/PR status means that the specimen is negative for both receptors. Nuc-pYStat5, nuclear localized and tyrosine phosphorylated Stat5; GLUT1, glucose transporter 1; PrlR, prolactin receptor; IDC, invasive ductal carcinoma; ILC, invasive lobular carcinoma; ER, estrogen receptor; PR, progesterone receptor.

When levels of GLUT1 expression within each cancer specimen were plotted against levels of Nuc-pYStat5, all tumors with elevated GLUT1 levels displayed low levels of Nuc-pYStat5, whereas all cases with high levels of Nuc-pYStat5 expressed low levels of GLUT1 (Figure 1B). This is consistent with mutually exclusive patterns of positive staining for GLUT1 and Nuc-pYStat5 at the global tumor level. However, since some Nuc-pYStat5-positive breast cancer cases display heterogeneous staining patterns with regional intratumoral loss of Nuc-pYStat5 (for example Figure 1C), we determined at a more refined scale whether mutually exclusive expression of GLUT1 and Nuc-pYStat5 also existed at the regional level within tumors. We co-stained and re-quantified levels of GLUT1 and Nuc-pYStat5 in the same 52 breast cancer specimens based on sampling of a total of 2,244 nonoverlapping 0.6 mm^2^ tumor regions. Also at this local scale, regions with high GLUT1 levels consistently displayed low Nuc-pYStat5 levels (Figure 1D, panel 1), whereas regions with high Nuc-pYStat5 intensities were associated with low GLUT1 levels (Figure 1D, panel 3). Partitioning of the sampled regions into either high or low GLUT1 or Nuc-pYStat5 levels revealed that areas with high GLUT1 levels had 1.72-fold elevated odds of displaying low Nuc-pYStat5 levels (odds ratio = 1.72, 95%CI: 1.12 to 2.66; *p* = 0.013).

Intriguingly, even among the sampled 2,244 tumor regions of 0.6 mm^2^ many still displayed heterogeneous positivity for either marker, but rarely did cells appear doubly positive for both markers (see representative image in Figure 1D, panel 2). We therefore further examined the relationship between GLUT1 and Nuc-pYStat5 levels within a subset of tumors exhibiting staining heterogeneity, and resolved marker expression at the single cell level using cell segmentation software. In total, images of six tumors co-stained for GLUT1 and Nuc-pYStat5 were analyzed to generate 8,804 cellular data points (Figure 1E, upper panels). A scatter plot of histocytometric GLUT1 and Nuc-pYStat5 values revealed continued mutually exclusive staining pattern between the two markers also at this scale (Figure 1E, lower panel). The odds of cells expressing low levels of Nuc-pYStat5 were 1.9-fold higher when the cells expressed high levels of GLUT1 (odds ratio = 1.9, 95% confidence interval (CI): 1.62 to 2.25; *p* <0.0001). Collectively, the quantitative analyses of clinical breast cancer specimens revealed a robust mutually exclusive pattern of expression between high GLUT1 and Nuc-pYStat5 levels, a relationship that persisted across all levels examined including at the global tumor tissue, locoregional, and cellular levels.

### Acidosis-induced disruption of all signals downstream of prolactin receptors

**Figure 2:**
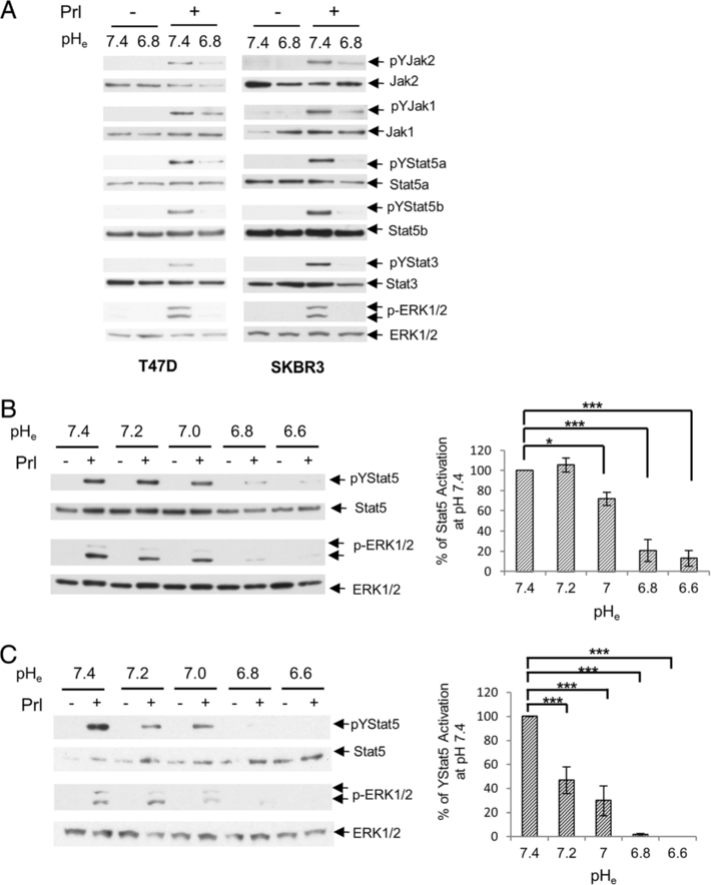
**Prolactin signaling in breast cancer cells is markedly inhibited by moderate acidic pH**_**e**_**. (A)** T47D cells and SKBR3 cells were induced by prolactin or vehicle at pH_e_ 7.4 or 6.8. All major prolactin signaling pathways were examined. T47D **(B)** and SKBR3 **(C)** cells were induced by prolactin or vehicle at various pH_e_ as indicated. Stat5 and Erk activation by prolactin was examined. Levels of Stat5 activation were analyzed by densitometry and plotted as mean ± SE (*n* = 3). The pYStat5 levels among different pH_e_ conditions were analyzed by ANOVA, and followed by Fisher’s least significant difference test. ANOVA, analysis of variance; pH_e_, extracellular pH; SE, standard error. *, *p* <0.05; **, *p* <0.01; ***, *p* <0.001.

Acidosis might affect prolactin-Stat5 signaling in breast cancer cells by mechanisms beyond disrupting prolactin receptor-ligand binding. However, if the principal mechanism involved is pH_e_-dependent disruption of receptor-ligand binding, then the effect on prolactin signaling would not be limited to Stat5 but include disruption of all signals downstream of the prolactin receptor. Indeed, in both T47D and SKBR3 cells all major prolactin-activated signaling pathways were inhibited at pH_e_ of 6.8, including inducible phosphorylation of Jak2, Jak1, signal transducer and activator of transcription-5a (Stat5a), signal transducer and activator of transcription-5b (Stat5b), Stat3 and Erk (Figure 2A). We next characterized the proton concentration-dependence of prolactin-responsiveness of breast cancer cells by analyzing prolactin-induced phosphorylation of Stat5 and Erk over a pathophysiological pH_e_ range from 7.4 to 6.6. Indeed, in both T47D (Figure 2B) and SKBR3 cells (Figure 2C), prolactin-induced Stat5 and Erk phosphorylation were gradually reduced with decreasing pH_e_. Prolactin-stimulated Stat5 and Erk phosphorylations in both cell lines were diminished by 80% or more at pH_e_ 6.8 compared with phosphorylation at pH_e_ 7.4 (*p* <0.01). Even at pH_e_ 7.0, only 0.4 pH units below normal tissue pH of 7.4, a statistically significant decrease in pYStat5 levels was detected. Prolactin signaling in SKBR3 cells was particularly sensitive to extracellular acidosis, possibly because prolactin receptor levels in SKBR3 cells are lower than in T47D cells.

### Acidosis-induced disruption of prolactin signaling is resistant to high prolactin concentrations

**Figure 3:**
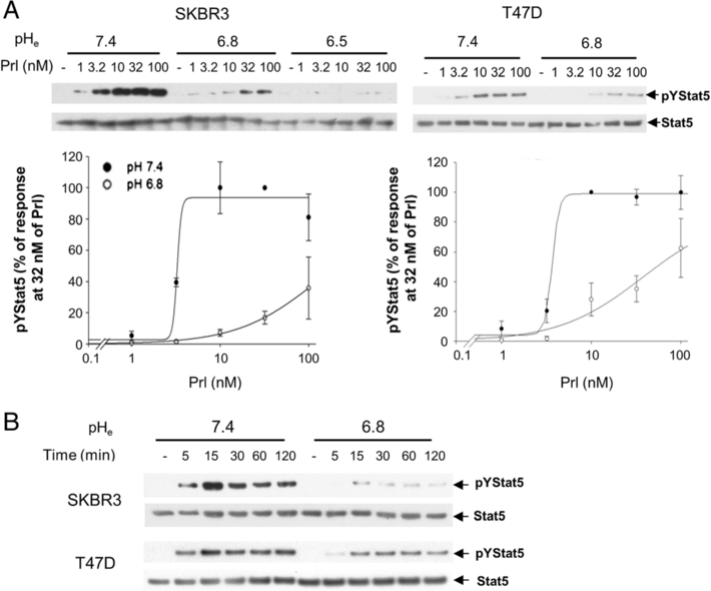
**Dose-dependent response and time course of prolactin-induced Stat5 tyrosine phosphorylation at different levels of pH**_**e**_**. (A)** Different concentrations of prolactin (1 to 100 nM) were used to induce SKBR3 or T47D cells at normal tissue pH_e_ (pH_e_ 7.4) or acidic tumor pH_e_ (pH_e_ 6.8 or pH_e_ 6.5). Representative data on levels of pYStat5 and total Stat5 protein is presented with densitometric analyses shown representing independent experiments in SKBR3 (*n* = 4) and T47D (*n* = 3). **(B)** SKBR3 and T47D cells were treated with prolactin for indicated length of time and representative pYStat5 and Stat5 levels are shown (*n* = 4). pH_e_, extracellular pH.

We next determined whether increasing concentrations of prolactin could overcome acidosis-suppression of prolactin signaling. SKBR3 and T47D breast cancer cell lines were stimulated with prolactin concentrations ranging from 1 to 100 nM for 15 min at different pH_e_. At pH_e_ of 7.4, Stat5 phosphorylation was detectably induced by 1 nM prolactin and reached maximum levels at 10 nM prolactin in both cell lines (Figure 3A). In contrast, in both cell lines at pH_e_ of 6.8, prolactin activation of Stat5 was greatly suppressed at all prolactin concentrations tested. SKBR3 cells were tested also at pH_e_ of 6.5, which completely abolished Stat5 activation even at 100 nM of prolactin. Estimated half maximal effective concentration (EC_50_) values were at least 10-fold higher at pH_e_ of 6.8 than at 7.4 (119.0 nM vs. 3.3 nM for SKBR3, 37.8 nM vs. 3.7 nM for T47D) (Figure 3B). Furthermore, a time course study up to 120 min eliminated the possibility that acidosis simply transiently suppressed and delayed prolactin receptor activation (Figure 3C). At pH_e_ of 7.4 prolactin-induced Stat5 activation peaked within 15 min, dropping slightly thereafter in both cell lines, whereas prolactin-induced pYStat5 levels were greatly diminished at pH_e_ 6.8, without evidence of emergence of a delayed signal. Together, these data established that prolactin signaling in human breast cancer cells is highly sensitive to extracellular acidosis even at high prolactin concentrations and for extended periods.

### Prolactin receptor signaling is selectively sensitive to the inhibitory effect of acidic microenvironment

**Figure 4:**
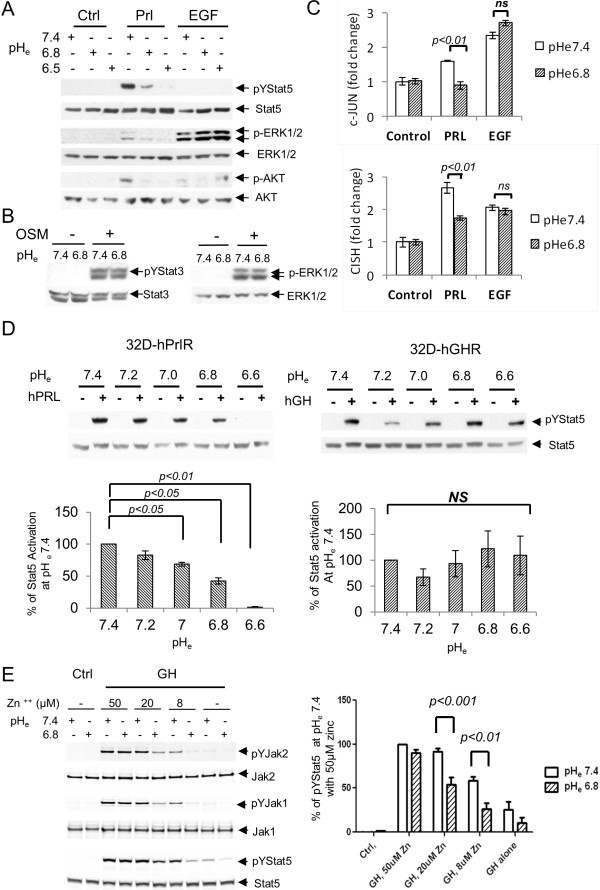
**pH**_**e **_**effect on prolactin receptor signaling is specific. (A)** SKBR3 cells were stimulated with vehicle, prolactin or EGF for 30 min at different pH_e_ as indicated. Stat5 and Erk phosphorylation were examined (*n* = 3). **(B)** SKBR3 cells were treated with vehicle or 10 nM OSM for 15 min at pH_e_ 7.4 or 6.8. Representative Stat3 and Erk activation were shown (*n* = 2). **(C)** SKBR3 cells were treated with vehicle, prolactin or EGF for 1 hour at pH_e_ 7.4 or 6.8. Quantitative PCR of *c-jun* and *CISH* transcripts were conducted and representative results are shown in bar graphs (*n* = 3). **(D)** 32D-hPrlR cells were treated with 2 nM prolactin at pH_e_ range of 7.4 to 6.6 (*n* = 3). 32D-hGHR cells were treated with 2 nM growth hormone at similar conditions (*n* = 5). Representative pYStat5 and total Stat5 blots are shown. Stat5 activation at each pH_e_ were analyzed and compared. **(E)**, 10 nM GH-induced phosphorylation of Jak2, Jak1 and Stat5 at various zinc concentration were compared at normal and acidic tumor pH_e_ in T47D cells (*n* = 4). One-way ANOVA was followed by Bonferroni’s post hoc test. 32D-hGHR, human growth hormone receptor cells; 32D-hPrlR cells, mouse promyeloid 32D cells stably transfected with human prolactin receptor; ANOVA, analysis of variance; EGF, epidermal growth factor; GH, human growth hormone; pH_e_, extracellular pH.

Since earlier reports had revealed a compromised solution structure of prolactin at lower pH [41] with reduced ability to bind to prolactin receptors in surface plasmon resonance assays [32], we examined whether the suppressive effect of acidic pH is selective for prolactin receptor signaling or reflects a general effect on cell surface receptor signaling in cancer cells stressed by exposure to low pH_e_. Whereas prolactin-induced phosphorylation of Stat5, Erk and Akt in SKBR3 cells were suppressed at pH_e_ of 6.8 and 6.5, Erk activation induced by EGF showed no evidence of inhibition by extracellular acidosis, compared to the response at normal pH_e_ of 7.4 (Figure 4A). Likewise, activation of Stat3 and Erk in SKBR3 cells by the proinflammatory cytokine, oncostatin M (OSM), was also unaffected by pH_e_ of 6.8 (Figure 4B). Similar insensitivity to moderate acidosis was observed for insulin-like growth factor 1 (IGF1) and fibroblast growth factor (FGF) signaling (see Additional file 1). Thus, under acidic conditions that completely blocked prolactin-induced signaling, breast cancer cells remained viable and fully capable of responding to other extracellular factors. Furthermore, while prolactin effectively induced both *c-jun* and *CISH* transcripts in SKBR3 cells at pH_e_ 7.4, transcript-inductions by prolactin but not EGF were markedly reduced at pH_e_ 6.8 (*P <0.01*) (Figure 4C).

Human growth hormone (GH) resembles prolactin in size and overall structure and is also implicated in breast cancer growth and development [42,43]. To examine whether GH receptor signaling is pH-dependent, we stably expressed GHR or hPrlR in the GHR and PrlR-negative 32D murine myeloblast line. We examined Stat5 activation in 32D-hPrlR or 32D-hGHR cells by the cognate ligands over a pH_e_ range of 7.4 to 6.6 (Figure 4D). Whereas prolactin-induced Stat5 phosphorylation was gradually lost with increasing proton concentrations in 32D-hPrlR cells, GH-induced Stat5 activation was not suppressed over the same pH_e_ range in 32D-hGHR cells (Figure 4D). These results demonstrated that GH signaling via GHR is resistant to extracellular acidosis.

Human PrlR does not only bind human prolactin, but is capable of binding and responding to GH, with zinc ions facilitating GH-hPrlR binding [44]. Many breast cancer cells express both GHR and PrlR. To test whether GH signaling in human T47D breast cancer cells, which express low levels of GHR and high levels of PrlR, is affected by extracellular acidosis, we assessed GH induction of phosphorylation of Jak2, Jak1 and Stat5 across a range of zinc ion concentrations at pH_e_ 7.4 and 6.8 (Figure 4E). Under zinc-free conditions GH signals in T47D cells were modest at normal pH_e_ and were suppressed at acidic pH_e_. Increasing concentrations of zinc ions enhanced GH signaling at pH_e_ 7.4, presumably by enhancing GH binding to PrlR. Since circulating zinc ion concentrations in humans range from 1 to 20 μM [45], only at supraphysiological zinc ion concentrations of 50 μM did GH-induced signaling in T47D cells become pH_e_-independent (Figure 4E). Collectively, our data indicates that at physiological zinc ion levels GH-induced Stat5 activation via PrlR, but not GHR, in breast cancer cells is significantly suppressed by moderate extracellular acidosis.

### Inhibition of prolactin signaling by acidosis is rapidly reversible

**Figure 5:**
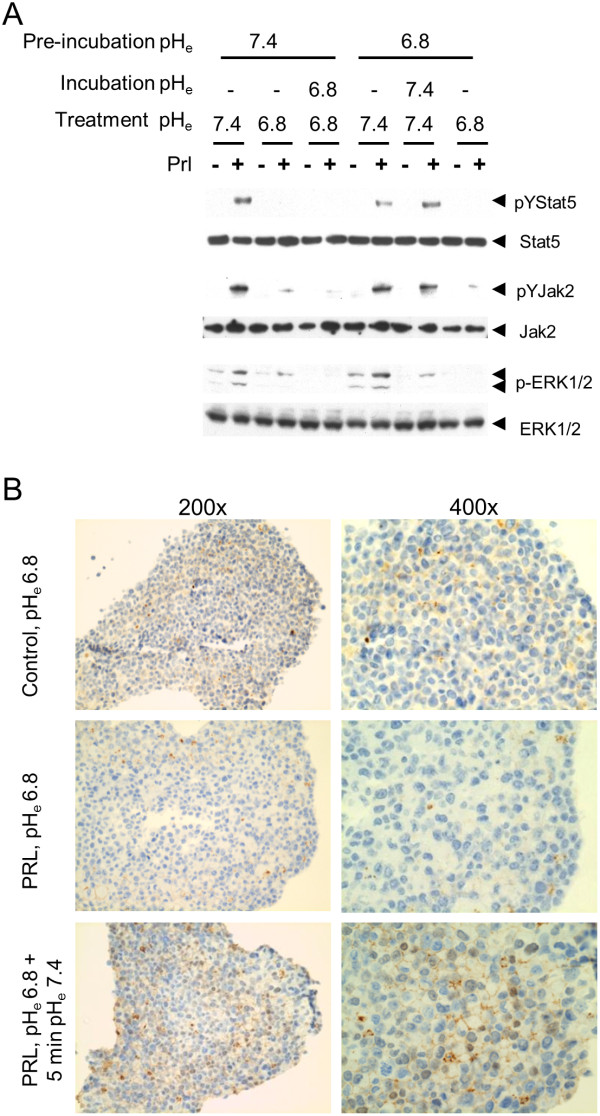
**pH**_**e **_**effect on prolactin signaling is rapidly reversible. (A)** SKBR3 cells were preincubated with medium of either pH_e_ 7.4 or pH_e_ 6.8 for 30 min at 37°C. Then the pH_e_ was altered or not as indicated and cells were treated with prolactin for 15 min. Two groups were incubated further for another 30 minutes as indicated before exposure to prolactin. Jak2, Stat5 and Erk activation was analyzed (*n* = 3). **(B)** T47D spheroids grown in three-dimensional culture conditions were treated with prolactin or vehicle at indicated pH conditions. The spheroids were stained for pYStat5. The experiment was carried out twice.

Our observation that all downstream signals are disrupted at reduced pH_e_ and data from cell-free analyses [32,33] suggest that proton-induced disruption of prolactin signaling occurs at the level of ligand-receptor binding. However, acidosis might indirectly affect prolactin receptor signaling through additional mechanisms. We therefore tested whether restoration of physiological pH_e_ could immediately rescue PrlR signaling, which would be consistent with a primary mechanism of direct disruption of ligand binding. SKBR3 cells were preincubated alternately at pH_e_ 7.4 or pH_e_ 6.8 for 30 min, before cells were exposed to medium containing prolactin or vehicle for 15 min at either the same pH_e_ or the alternate pH_e_, and prolactin-induced Stat5, Jak2 and Erk signals were examined. A third set of cells received additional 30 min incubation in the alternate pH_e_ before prolactin exposure to determine whether prolonged reversal of pH_e_ would be more effective than immediate reversal (Figure 5A). For cells preincubated at pH 7.4, the inhibition of prolactin signals by pH_e_ 6.8 was immediate and not further strengthened by additional incubation at pH_e_ 6.8. Likewise, rescue of prolactin signaling in pH_e_ 6.8-exposed cells was immediately restored upon exposure to prolactin at pH_e_ 7.4, and signals were not consistently further enhanced by prolonged incubation at pH_e_ 7.4 prior to prolactin stimulation.

To better experimentally model prolactin-acidosis relationships in tumors *in vivo*, we used three-dimensional T47D spheroids in culture to determine whether acidosis-induced suppression of prolactin signaling could be rapidly reversed by normalizing pH_e._ Indeed, when three-dimensional spheroids of T47D cultures at pH_e_ 6.8 were equilibrated with prolactin for 2 h there was no detectable Stat5 activation over control levels, whereas alkalinization of the medium to pH 7.4 restored Stat5 activation within 5 min in prolactin-equilibrated spheroids (Figure 5B). Collectively, extracellular acidosis inhibits prolactin signaling in a rapid and reversible manner consistent with the principal mechanism being proton-dependent disruption of ligand-receptor binding.

### Markers of glucose uptake/glycolytic metabolism are associated with loss of Nuc-pYStat5 signaling in invasive breast cancer and xenografts in mice

**Figure 6:**
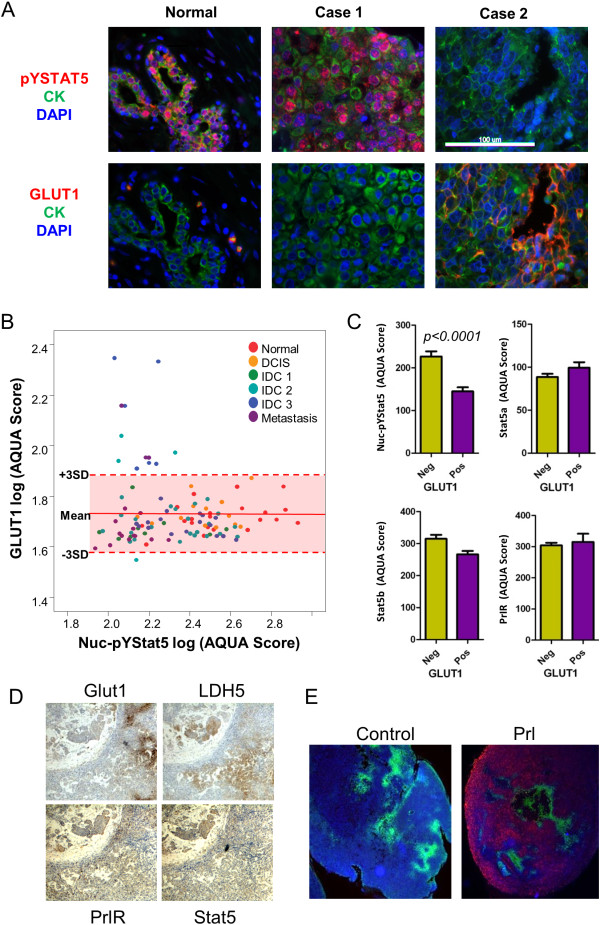
**Elevated GLUT1 is associated with low Nuc-pYStat5 in human breast cancer and xenografts. (A)** Representative images from a breast cancer progression tissue array co-stained with anti-cytokeratins, DAPI, anti-pYStat5 or anti-GLUT1. **(B)** Expression of GLUT1 and nuclear pY-Stat5 in each tissue samples were quantified by AQUA and plotted. The range of normal GLUT1 expression is labeled on the plot by mean of normal mammary gland GLUT1 (log) score ± 3 SD. **(C)** Comparison of AQUA scores Nuc-pYStat5, Stat5a, Stat5b and PrlR between GLUT1 negative and positive breast cancer samples (*n* = 88) by Student’s *t* test. **(D)** IHC staining of LDH5, GLUT1, PrlR and Stat5 with consecutive slides of a representative T47D xenograft tumor. **(E)** Co-staining of pYStat5 and GLUT1 in representative T47D xenografts from mice that were injected intraperitoneally with vehicle or human prolactin. Red, pYStat5; green, GLUT1. AQUA, automated quantitative analysis; GLUT1, glucose transporter 1; IHC, immunohistchemistry; LDH5, lactate dehydrogenase-5; Nuc-pYStat5, nuclear localized and tyrosine phosphorylated Stat5; PrlR, prolactin receptor; SD, standard deviation.

To further substantiate the observed mutually exclusive expression of the glucose transporter GLUT1 and Nuc-pYStat5 in tissue sections from Cohort I of clinical breast cancer cases, we examined a larger Cohort II of archival breast cancer tissues that also included normal controls. Normal breast epithelia generally displayed high levels of Nuc-pYStat5 and low levels of GLUT1. Representative tissue sections of normal breast tissue stained for either pYStat5 or GLUT1 along with two cases of invasive breast cancer that are positive for either Nuc-pYStat5 or GLUT1 are shown (Figure 6A). Levels of GLUT1 and Nuc-pYStat5 within the epithelial compartment were quantified for each specimen of Cohort II using AQUA and presented as a scatter plot stratified by diagnostic categories (Figure 6B). GLUT1 membrane staining was generally undetectable in normal breast epithelia but was elevated in a subset of invasive breast carcinomas (Figure 6B). Consistent with previous reports [27,38], Nuc-pYStat5 levels were high in the epithelia of healthy breast tissues and reduced in many of the invasive ductal carcinomas (IDCs). GLUT1-positive breast cancer cases not only displayed reduced Nuc-pYStat5 staining, but also represented more progressive disease, such as higher grade of cancer or lymph node metastases. Among malignant breast specimens, levels of Nuc-pYStat5 were substantially lower in GLUT1-positive cases than in GLUT1-negative cases (*p* <0.001), whereas expression levels of Stat5a, Stat5b and PrlR did not differ between the two groups (Figure 6C). We further partitioned malignant breast cancer specimens into low and high Nuc-pYStat5 based on the range in healthy breast tissues. Consistent with the data of Cohort I of 52 invasive breast cancer specimens, high GLUT1 levels were associated with low Nuc-pYStat5 levels in the invasive breast cancer specimens of Cohort II (N = 88; *p* = 0.01, Fisher’s exact test).

We further detected regional co-expression of GLUT1 and the key glycolytic enzyme, lactate dehydrogenase-5 (LDH5), in T47D xenotransplants indicative of localized glycolytic metabolism and lactic acid production within the tumors (Figure 6D). In contrast, expression of PrlR and Stat5 proteins were largely uniform within the xenotransplants, regardless of glycolytic markers. We therefore hypothesized that GLUT1-positive T47D tumor regions would be resistant to exogenous human prolactin. Consistent with local acidosis within T47D tumors, *in vivo* pH measurement by microelectrode probe revealed a variably acidic tumor interstitium averaging pH_e_ of 6.61 ± 0.11 (*n* = 9), significantly lower than pH_e_ of mouse peritoneal cavity at 7.25 ± 0.04 (*n* = 3; *p* <0.001 by Student’s *t* test). We injected human prolactin or vehicle into nude mice bearing T47D human xenografts and collected tumors 1 h later. When tumor sections were co-stained for pYStat5 and GLUT1, all T47D xenografts were regionally positive for GLUT1 staining (Figure 6E). Consistent with local acidosis-mediated suppression of prolactin signaling, GLUT1-positive regions as well as the immediately surrounding zone lacked prolactin-inducible pYStat5 response whereas regions of GLUT1-negative tumor cells displayed robust pYStat5 signal (Figure 6E). These *in vivo* experiments confirmed the presence of interstitial acidosis and regionally increased glucose uptake and glycolytic metabolism in T47D xenotransplants, and established selective unresponsiveness of GLUT1-positive tumor regions to exogenous human prolactin.

## Discussion

The present study supports the novel pathophysiological concept that extracellular acidosis within the microenvironment of breast cancer potently and selectively disrupts prolactin receptor signaling, including Stat5 activation. Previous analyses of more than 2,000 cases revealed that loss of nuclear translocated and tyrosine phosphorylated Stat5 (Nuc-pYStat5) occurs frequently in breast cancer, and correlates with disease progression, poor prognosis, and increased risk of resistance to endocrine therapy [27-30]. Intratumoral acidosis is a previously unrecognized factor contributing to loss of prolactin-induced Nuc-pYStat5 human breast cancer, and implicates acidosis-associated prolactin resistance as a novel mechanism by which breast cancer cells escape pro-differentiation and invasion-suppressive effects of prolactin.

The pathophysiological relevance of potent and reversible acidosis-disruption of prolactin signaling in breast cancer is supported by extensive experimental evidence and correlative studies in archival human breast cancer specimens provided clinical relevance. Indeed, we observed mutually exclusive expression patterns of Nuc-pYStat5, a marker of prolactin receptor activation, and elevated levels of GLUT1, a marker of increased glycolysis and associated lactacidosis. Quantitative multiplexed immunofluorescence analyses specifically revealed that positive GLUT1 expression (gain-of-function) was associated with low levels of Nuc-pYStat5 (loss-of-function) in malignant breast tumors at three different scales: at the global tumor level, regionally within tumors, and at the cellular level. This is consistent with global and regional acidosis within malignant breast tumors. However, a substantial number of tumors, tumor regions, or tumor cells were negative for both GLUT1 and Nuc-pYStat5, indicating that not all tumor-associated absence of Stat5 signaling is explainable by GLUT1-associated acidosis in breast carcinoma cells. For instance, acidosis may in some cases be caused by increased glycolysis within stromal fibroblasts [6], which is not correlated with epithelial GLUT1 staining. Furthermore, alternative mechanisms likely lead to loss of Nuc-pYStat5 in human breast cancer, such as inhibition of prolactin-Stat5 signaling by the tyrosine phosphatase PTP1B through inhibition of the Jak2 tyrosine kinase [46].

Aerobic glycolysis in carcinoma cells is frequently associated with activated oncogenes, including Src, Myc, AKT/mTOR pathway and mutation of tumor suppressors such as p53 [47]. In these cases, the entire tumor typically displays glycolytic metabolism regardless of oxygenation status. In fact, we observed that more than half of the GLUT1-positive human breast cancer specimens displayed generally homogenous GLUT1 staining throughout the tumor and were essentially negative for Nuc-pYStat5. Alternatively, rapidly proliferating tumors may exhibit regional hypoxia with resulting focal or regional hypoxia-induced glycolysis and acidosis. Indeed, heterogeneous GLUT1 staining in breast tumors was also commonly detected, including specimens with GLUT1-positive foci surrounding necrotic regions suggestive of local hypoxia. More recently, paracrine hepatocyte growth factor from cancer-associated fibroblasts was shown to promote GLUT1 expression and the Warburg effect in cancer [48]. Regardless of the mechanisms underlying increased regional glucose metabolism and extracellular acidosis, carcinoma cells positive for Nuc-pYStat5 were absent in tumor regions where carcinoma cells displayed elevated GLUT1. Experimental evidence for acidosis-induced suppression of prolactin signaling in breast cancer was extended from cell lines in two-dimensional cultures to human breast cancer xenotransplants in mice *in vivo* and to multilayered three-dimensional spheroid cultures, experimental conditions that better mimic local acidosis within the patient tumor microenvironment. In fact, T47D xenotransplant tumor regions expressing high GLUT1 were resistant to exogenous prolactin despite retaining prolactin receptor and Stat5 expression. Furthermore, in three-dimensional spheroids of T47D cells extracellular alkalinization alone rapidly reversed acidosis-disrupted prolactin signaling. These observations are consistent with the notion that elevated glycolysis and lactacidosis effectively disrupts prolactin-induced Nuc-pYStat5 in breast cancer.

The sensitivity of prolactin-induced signaling to acidosis is most likely due to a mechanism that involves protonation of histidine residues located at the ligand-receptor binding interface [33,49]. Four histidine residues are directly involved in high-affinity binding between prolactin and its cognate receptor based on crystal structures [41]. In contrast, histidine residues are not critical for binding of the closely related but acidosis-resistant growth hormone to its cognate GHR [50]. Mutational analyses have suggested that H180 of prolactin and H188 of PrlR are particularly important for the pH-dependent ligand-receptor binding. Importantly, human GH can also bind to hPrlRs and exert lactogenic activity [51]. The binding of GH to hPrlRs is facilitated by a critical Zn^2+^ binding site formed by two growth hormone residues (H18 and E174) and two prolactin receptor residues (D187 and H188) at the binding interface [52]. Therefore, protonation of prolactin receptor H188 at acidic pH may interfere with Zn^2+^-mediated binding of GH, and consequently disrupt the majority of GH-induced PrlR signaling in T47D cells. Only in the presence of supraphysiological concentrations of Zn^2+^ (50 μM) did GH-induced signaling become resistant to acidosis in T47D cells. This effect is probably due to stabilization of the histidine imidazole group at high Zn^2+^ concentration, which might protect PrlR H188 from becoming protonated. These observations are consistent with an earlier report based on a cell-free assay that the binding of GH to the PrlR extracellular domain was not pH-dependent in the presence high levels of Zn^2+ ^[33]. Importantly, GH signaling through PrlRs, including proposed heterodimerization of PrlRs and GHRs [53], is likely to remain pH_e_-dependent at physiologic Zn^2+^ levels. In contrast, GH activation of GHRs expected to be unaffected by acidosis.

In addition to the full-length or ‘long’ PrlR, alternative mRNA splicing generates ‘intermediate’ and ‘short’ PrlR isoforms that only differ in their cytoplasmic domain. Binding of prolactin to these PrlR isoforms is expected to remain sensitive to acidosis. On the other hand, binding of prolactin to the ΔS1 PrlR isoform may be less sensitive to acidosis due to its already poor ligand-binding kinetics caused by partial loss of the ligand-binding interface [54]. Furthermore, 16 K prolactin is an N-terminal proteolytic fragment of prolactin that comprises only the first 145 amino acid residues. Therefore, 16 K prolactin lacks the critical H180 residue which mediates pH_e_-sensitive binding of Prl to PrlR. Despite the well-documented anti-angiogenesis activity of 16 K prolactin, the receptor mediating its function remains to be identified, and the effect of acidosis on the function of 16 K prolactin is unknown. Interestingly, 16 K prolactin is generated by cathepsin D cleavage of full length prolactin [55]. Cathepsin D is a lysosomal protease and thought to be active only at lysosomal pH range (approximately 5.0). More recent studies suggested that cathepsin D could be secreted and activated at acidic pH_e_ approximately 6.7 [56]. Therefore, an acidic tumor environment might facilitate cleavage of full length prolactin into 16 K prolactin.

## Conclusions

In summary, the prolactin-Jak2-Stat5 pathway, which may suppress breast cancer cell epithelial-to-mesenchymal transition, invasion and drug resistance [21-23], is potently and selectively suppressed by extracellular tumor acidosis. Local acidosis within the breast cancer microenvironment may represent a significant contributor to loss of Nuc-pYStat5 detected in clinical breast cancer specimens and thereby promote progression and evolution of more invasive and therapy-resistant disease. Studies are warranted to determine how extracellular tumor acidosis impacts pharmacological strategies centered on targeting prolactin receptor pathways [57,58] in breast cancer and potentially other malignancies.

## Abbreviations

pHe: extracellular pH; 32D-hGHR: human growth hormone receptor cells; 32D-hPrlR cells: mouse promyeloid 32D cells stably transfected with human PrlR; ANOVA: analysis of variance; AQUA: automated quantitative analysis; CI: confidence interval; DAPI: 4′,6-diamidino-2-phenylindole; EC50: half maximal effective concentration; EGF: epidermal growth factor; ER: estrogen receptor; FCS: fetal calf serum; FGF: fibroblast growth factor; hGH: human growth hormone; hGHR: human growth hormone receptor; GLUT1: glucose transporter 1; hPrlR: human prolactin receptors; IDC: invasive ductal carcinoma; IGF1: insulin-like growth factor 1; IHC: immunohistochemistry; LDH5: lactate dehydrogenase-5; Nuc-pYStat5: nuclear localized and tyrosine phosphorylated Stat5; OSM: oncostatin M; pHe: extracellular pH; PR: progesterone receptor; SE: standard error; Stat5a: signal transducer and activator of transcription-5a; Stat5b: signal transducer and activator of transcription-5b.

## Competing interests

The authors declare that they have no competing interests.

## Authors’ contributions

NY, ARP, CL, TT, BF, IC, TH, CDS, JAH, AJK and HR conceived the study and participated in its design. JAH, AJK, CDS, THT and HR provided formalin-fixed, paraffin-embedded archived patient materials for the study. CL, MAG and ARP performed immunostaining, and quantitative immunofluorescence analyses. JAH, AJK and CDS conducted pathologic reviews and clinical data evaluations. ARP, BF, IC, TH, NY, ARP and HR performed statistical analyses. TT cultured three-dimensional T47D spheroids. FEU generated 32D-hPrlR and 32D-hGHR cell lines. AFY and NY conducted *in vivo* and *in vitro* experiments. NY and HR drafted the manuscript. All authors read, edited and approved the final manuscript.

## Supplementary Material

Additional file 1 **FGF and IGF1 signaling was not affected by acidic pH**_**e**_**.** SKBR3 cells were treated with FGF (20 ng/ml) or IGF1 (100 ng/ml) for 15 min at either pH_e_ 7.4 or 6.8. Representive immunoblots of pErk1/2 and Erk are shown (n = 3). FGF, fibroblast growth factor; IGF1, insulin-like growth factor 1; pH_e_, extracellular pH. Click here for file
