# Supplementary figures and images for: Prolactin-Stat5 signaling in breast cancer is potently disrupted by acidosis within the tumor microenvironment
Source: Breast Cancer Res. 2013 Sep 3;15(5):R73. doi: 10.1186/bcr3467 (PMC3978581; doi:10.1186/bcr3467)

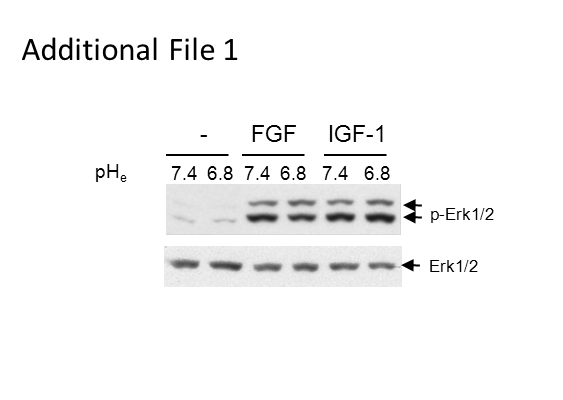

Supplement: Additional file 1 — FGF and IGF1 signaling was not affected by acidic pHe. SKBR3 cells were treated with FGF (20 ng/ml) or IGF1 (100 ng/ml) for 15 min at either pHe 7.4 or 6.8. Representive immunoblots of pErk1/2 and Erk are shown (n = 3). FGF, fibroblast growth factor; IGF1, insulin-like growth factor 1; pHe, extracellular pH. [file bcr3467-S1.tiff]
